# Real-Time and Fully Automated Robotic Stacking System with Deep Learning-Based Visual Perception

**DOI:** 10.3390/s25226960

**Published:** 2025-11-14

**Authors:** Ali Sait Ozer, Ilkay Cinar

**Affiliations:** 1 Department of Control and Automation Technology, Konya Technical University, 42250 Konya, Türkiye; asozer@ktun.edu.tr; 2 Department of Computer Engineering, Selcuk University, 42250 Konya, Türkiye

**Keywords:** computer vision, industrial automation, programmable logic controller integration, real-time object detection, robotic stacking, smart manufacturing

## Abstract

**Highlights:**

The proposed framework represents a fully deployable AI-driven automation system that enhances operational accuracy, flexibility, and efficiency. It establishes a benchmark for smart manufacturing solutions that integrate machine vision, robotics, and industrial communication technologies. The study contributes to the advancement of Industry 4.0 practices by validating an intelligent production model applicable to real industrial environments.

**What are the main findings?**

- A real-time image processing framework was developed in Python using the YOLOv5 models and directly integrated into an industrial production line.
- The system successfully combined object classification results with a Siemens S7-1200 PLC via Profinet communication, enabling synchronized control of the robotic arm, conveyor motors, and sensors.

**What are the implications of the main findings?**

- The integration of deep learning-based visual perception with PLC-controlled automation enables seamless communication between vision and mechanical components in industrial settings.
- The validated framework demonstrates scalability and real-world applicability, offering an effective solution for multi-class object detection and robotic stacking in manufacturing environments.

**Abstract:**

This study presents a fully automated, real-time robotic stacking system based on deep learning-driven visual perception, designed to optimize classification and handling tasks on industrial production lines. The proposed system integrates a YOLOv5s-based object detection algorithm with an ABB IRB6640 robotic arm via a programmable logic controller and the Profinet communication protocol. Using a camera mounted above a conveyor belt and a Python-based interface, 13 different types of industrial bags were classified and sorted. The trained model achieved a high validation performance with an mAP@0.5 score of 0.99 and demonstrated 99.08% classification accuracy in initial field tests. Following environmental and mechanical optimizations, such as adjustments to lighting, camera angle, and cylinder alignment, the system reached 100% operational accuracy during real-world applications involving 9600 packages over five days. With an average cycle time of 10–11 s, the system supports a processing capacity of up to six items per minute, exhibiting robustness, adaptability, and real-time performance. This integration of computer vision, robotics, and industrial automation offers a scalable solution for future smart manufacturing applications.

## 1. Introduction

In industrial production processes, the importance of parameters such as flexibility, speed, and quality is steadily increasing, necessitating the support of production lines with smarter and more autonomous systems [1,2]. In particular, labor-intensive solutions still widely used for repetitive tasks such as classification, sorting, and stacking often lead to time losses and inconsistencies in quality control processes. To address these issues, the integration of image processing and artificial intelligence-based decision-making systems into production workflows has gained significant momentum [3].

Image processing techniques enable the differentiation of products by analyzing visual features such as shape, color, text, and logos, thereby surpassing the capabilities of traditional sensor-based approaches [4]. Among these techniques, the YOLO (You Only Look Once) family is widely used in real-time object recognition tasks and is favored in industrial applications due to its low latency and high accuracy. In this context, the YOLOv5 model, with its high processing speed, ensures reliable classification on dynamic production lines [5].

However, the success of image processing algorithms alone is not sufficient in industrial environments. Seamless integration of these systems with industrial automation components is crucial to ensure real-time and stable operation [1]. In this regard, Programmable Logic Controllers (PLCs) play a key role by processing data received from image processing units and coordinating the control of robotic arms, conveyor systems, and other actuators in a synchronized manner [6]. Moreover, the simultaneous operation of robotic arms with these systems is essential for the precise positioning and stacking of products without error.

In this study, a real-time and fully integrated automation system was developed to automatically classify randomly moving bags on a conveyor belt using image processing and to stack them into predefined areas with an ABB IRB6640 industrial robotic arm (ABB Robotics, İstanbul, Türkiye). Thirteen types of industrial bags (kraft paper bags), each with varying colors and patterns, are placed randomly on the conveyor line. The products are captured by a camera mounted above the conveyor. The image processing workflow is executed by an interface software developed in Python, through which each bag is classified using the YOLOv5s model.

The identified class ID is directly transmitted to a Siemens S7-1200 series PLC via a communication protocol. The PLC processes this information and sends corresponding commands to the ABB robotic arm through the Profinet interface. The robotic arm then picks the classified bag from its position on the conveyor and places it in the appropriate stacking area. Once the operation is complete, it notifies the PLC, which concludes the cycle and restarts the conveyor to allow the next item to enter the system.

Unlike previous studies, this work contributes a real-time, end-to-end integration framework that connects a YOLOv5s perception model, a PLC communication channel, and a robotic arm through synchronized Profinet signaling. The originality of this study lies in demonstrating how these components can operate autonomously and reliably in an actual industrial production line and even obtain a better outcome also bridging the gap between laboratory research and field deployment.

The developed system features a dynamic and flexible structure that allows up to three different product types to be stacked simultaneously. Although the number of predefined product classes is 13, the system is modularly designed to accommodate additional classes with ease. By ensuring the seamless coordination of all components, the system establishes a high-speed, low-error production infrastructure that requires minimal human intervention.

The primary objective of this study is to present an integrated system design that combines image processing, PLC control, industrial communication protocols, and robotic motion control. By doing so, it aims to enhance the level of automation in repetitive production tasks such as classification and stacking, and to demonstrate the system’s applicability under real-world industrial conditions. The proposed system offers a scalable, adaptable, and highly accurate solution that serves as a valuable reference for future automation technologies.

## 2. Related Works

In recent years, the integration of deep learning-based image processing algorithms into industrial automation systems has become a significant area of research. In this context, various studies have been conducted to perform repetitive tasks on production lines. Among these tasks are classification, quality control, sorting, and stacking, which they use autonomous systems for. Below are some prominent examples of these studies.

Byambasuren et al. (2020) [7] developed a system that combines image processing techniques with industrial robot technology to automate quality control in manufacturing processes. Using an ABB IRB 120 industrial robot arm, they aimed to distinguish between normal and defective products through a quality assurance process. Instead of real products, 3D objects representing features such as color, fullness, and shape were used. The image processing stages were implemented using the OpenCV library, applying image acquisition, enhancement, thresholding, segmentation, and object recognition. The HSV color space was used to improve robustness against lighting variations. Based on the image analysis results, the system identified defective items and directed the robot arm to relocate them accordingly. The study concluded that the algorithm successfully distinguished between normal and faulty products, with the robot arm effectively executing physical sorting. It was suggested that the system could be integrated into real factory environments for high-precision applications in the future.

Mao et al. (2022) [8] developed a system that combines deep learning algorithms with a robotic arm to detect surface defects on aluminum wheels. In the study, defects such as dirt, paint stains, scratches, and dents were identified using YOLO-based models applied to images captured by a camera mounted on an ABB robotic arm. To enhance the dataset, GAN and DCGAN architectures were used, and the system was tested in the RobotStudio simulation environment. According to the simulation results, the best performance for the YOLOv4 model supported by synthetic data achieved a mAP of 84.0%. However, it was noted that the system was not implemented in a real production environment and was only tested virtually without integration of the robot, image processing, and PLC structure.

Rybczak et al. (2022) [9] aimed to develop an industrial automation system by integrating artificial intelligence (AI), image processing, and PLC technologies. In their study, images captured by a camera were processed using OpenCV, and an artificial neural network (ANN) was trained for object classification. The results were physically implemented using an ABB IRB 120 robotic arm. The experimental dataset consisted of objects with various colors and shapes. The developed model achieved over 90% accuracy in classifying objects and successfully directed the robot arm with correct commands. Based on classification results, objects were sorted through robot movement commands. System control was managed using a Siemens PLC, with data exchange between the AI model and PLC facilitated by Python-based middleware. The authors concluded that this integration can enhance flexibility and accuracy in production lines.

Kijdech and Vongbunyong (2023) [10] developed a real-time pick-and-place system using the ABB YuMi collaborative robot and an RGB-D camera. Object detection was performed using the YOLOv5 algorithm, incorporating both color and depth information. The system was tested under three scenarios: with a pre-trained model, an untrained model, and real-time camera input. These tests yielded accuracy rates of 95%, 90%, and 90%, respectively. Additionally, the model demonstrated high performance with precision (100%), recall (100%), mAP@0.5 (100%), and mAP@0.5:0.95 (90%). While the study showed strong results in object detection and positioning, it did not include integration with conveyor systems, PLC control, or industrial automation scenarios.

Shaloo et al. (2024) [11] developed a YOLOv8-based quality control system, offering a low-cost solution for real-time detection of correct and defective parts on an assembly line. Using software developed with Python, Siemens TIA Portal, and Mitsubishi RT Toolbox 2, the system performed image-based classification and enabled bidirectional communication between the PLC and the robotic arm. The system achieved a classification accuracy of 98% and was highlighted as particularly suitable for small and medium-sized enterprises (SMEs).

Wang et al. (2024) [12] developed a YOLOv7-based image processing system and presented a prototype that uses an ABB IRB120 robotic arm to sort soft plastic bags in real time on a moving conveyor. The system, integrated with a depth camera, the SORT algorithm, and the RAPID programming language, achieved a 96% mAP and operated with a response time measured in seconds.

Kirda et al. (2024) [13] developed a system integrating the YOLOv5 algorithm, an NVIDIA Jetson Nano microcontroller, and a Mitsubishi RV-2F-1D1-S15 robotic arm to automate the detection and processing of metal edges. The study focused on identifying surface defects such as sharp edges, burrs, and chamfers. The YOLOv5 model was compared with common edge detection algorithms like VGG-16 and ResNet, achieving the highest performance with a mean average precision (mAP) of 95.7%. The defect information detected by the image processing model was transmitted in real time to the robot control unit, enabling automated edge refinement operations.

Sayem et al. (2024) [14] developed a deep learning-based system aimed at improving efficiency in waste management by classifying and detecting urban solid waste. The study used a dataset of 10,406 images covering 28 categories of recyclable waste. A dual-stream convolutional neural network (CNN) architecture was proposed for classification, achieving an overall accuracy of 83.11%. For object detection, the GELAN-E (Generalized Efficient Layer Aggregation Network) model was used, yielding a mean mAP@50 of 63%. While the study presents a scalable and sustainable approach for developing automatic waste sorting systems in resource-limited settings, it does not include integration with industrial components such as robotic hardware, conveyor systems, or PLCs.

Bueno et al. (2024) [15] developed a ROS 2-based modular integration framework aimed at enhancing the flexibility and task adaptability of robotic manipulators for industrial and collaborative robotics applications. The framework was tested on an ABB IRB-120 robot equipped with a YOLOv8 object detection model and an OpenCV-based pose estimation module. In a cube pick-and-place scenario, the system achieved 100% accuracy in object detection and pose estimation in simulation and 97.43% object detection accuracy with 98.7% pose estimation accuracy in 78 real-world trials. Additionally, over 2600 robotic motions (Move and RobMove) were executed via ROS 2, with only 5 motion-related errors reported. The system’s overall performance was rated as “excellent” at 74% in simulation and 74.36% in real-world settings, depending on the robot hardware.

Almtireen et al. (2025) [16] developed a smart conveyor prototype equipped with a YOLOv8-based AI vision system controlled by a PLC, successfully classifying metal, plastic, and paper waste. While the AI model handled object recognition, the PLC managed actuators and conveyor control. Experimental results demonstrated the system’s effectiveness, achieving 90% overall accuracy and an average precision (mAP@50) of 86%.

Malobický et al. (2025) [17] developed a computer vision-based control system to enhance intuitive human–robot interaction. Using the ABB YuMi dual-arm collaborative robot, they designed an autonomous station that hands tools to operators. The system integrates YOLOv7 and YOLOv8 for recognizing both hand gestures and tools on the work surface. For gesture recognition, 13 static hand gestures from the HaGRID dataset were used, while a custom synthetic dataset was created for tool detection. YOLOv8 outperformed in detecting tools under various rotations but showed limited segmentation accuracy compared to YOLACT. In gesture recognition, YOLOv7 performed better, especially with limited training data. The system enables tool selection through hand gestures, identifies the tool’s position and orientation, and allows the robot to deliver it accurately. Results showed that integrating visual data with robot control enhanced human–robot collaboration and offered an intuitive user experience in production environments. The system is real-time, modular, and has strong potential to improve ergonomics on factory floors.

Kır et al. (2025) [18] developed a defect detection robotic cell that combines a YOLOv5-based deep learning algorithm with image processing techniques for quality control of automotive parts. The system, designed specifically to evaluate complex structures such as bolt thread geometry, achieved a 97.4% success rate (mAP) using a custom dataset of 3500 images. While the system offers high accuracy in visual defect detection, it does not provide detailed information regarding the integration of robotic arms, PLCs, or conveyor systems.

Nguyen et al. (2025) [19] developed a pick-and-place control system for industrial robots based on eye-hand coordination, using a cost-effective 2D camera and YOLOv7-GAN-based object recognition algorithms. The system operated at a speed of 220–250 products per hour, with GAN-augmented images used during model training. This approach achieved 83.41% accuracy with YOLOv5 and 94% with YOLOv7. The system was tested in both simulation and real environments, demonstrating high accuracy in object positioning and pick-and-place tasks using low-cost sensors.

Simeth et al. (2025) [20] aimed to develop a flexible and reliable object detection system for use on production lines. They designed a system capable of detecting parts with varying geometries and colors, integrating a YOLOv5-based deep learning approach with OpenCV-based image processing algorithms. The system was developed in Python to operate synchronously with a robotic arm and a Siemens S7-1500 PLC. Training data were collected from image sets of various industrial object types, and the system was tested under both artificial and real environmental conditions. The YOLOv5 model achieved a precision of 96.2%, recall of 93.5%, and an mAP@0.5 score of 95.1%. The authors reported that the model performed well under challenging lighting, diverse backgrounds, and object variations, suggesting its effectiveness for quality control and autonomous production processes.

Sokolov et al. (2025) [21] aimed to enhance human–robot collaboration by developing an intelligence-driven interaction system. In their study, an AI system capable of processing visual and auditory sensory data was integrated with an ABB IRB 120 robotic arm. YOLOv5 was used for visual recognition, while open-source speech processing algorithms handled voice recognition. The entire system was programmed in Python, with communication between the PLC and robot managed via ROS (Robot Operating System). Test data included videos and audio recordings featuring various command scenarios and object combinations. Experiments reported a 94% accuracy in object recognition and a 91% success rate in responding to voice commands. The authors concluded that the proposed system can support safe and effective human–machine interaction in flexible production lines.

A review of the literature shows that the integration of image processing algorithms, robotic control, and artificial intelligence-based systems into production lines has made significant contributions. Shaloo et al. [11] developed a YOLOv8-based quality control system establishing bidirectional communication between the PLC and the robotic arm but tested their system in an experimental setup rather than a real production environment. Similarly, Wang et al. [12] presented a prototype using the ABB IRB120 robot, but due to limited hardware scale and controlled conditions, the application remained constrained. Almtireen et al. [16] achieved 90% accuracy with a PLC-supported conveyor system, yet their system did not include industrial robot integration. Although Kır et al. [18] successfully applied image processing and deep learning algorithms, their system was not fully integrated with production line components (PLC, robot, conveyor). Mao et al. [8] and Kijdech & Vongbunyong [10] tested their robotic and vision systems solely in simulation environments, excluding practical challenges of real-world production settings. Nguyen et al. [19] reported effective results with low-cost hardware, but did not present a comprehensive structure for PLC and production line integration. Kirda et al.’s [13] system performed real-time robotic correction tasks, but was not fully integrated into a production line scenario. Sayem et al. [14] focused on classification performance but did not provide a full-scale implementation with industrial components. In contrast, the system developed in this study is one of the examples where a YOLOv5-based image processing model, Siemens S7-1200 PLC (Siemens Sanayi ve Ticaret A.Ş., İstanbul, Türkiye), ABB IRB6640 robotic arm, and conveyor system are fully integrated and operated on a real production line, achieving 100% stacking accuracy in a field test involving 9600 bags. This holistic integration stands out in literature not only for its theoretical success but also as a practical solution offering higher reliability, accuracy, and scalability in industrial environments.

## 3. Materials and Methods

### 3.1. System Hardware Configuration

**Figure 1:**
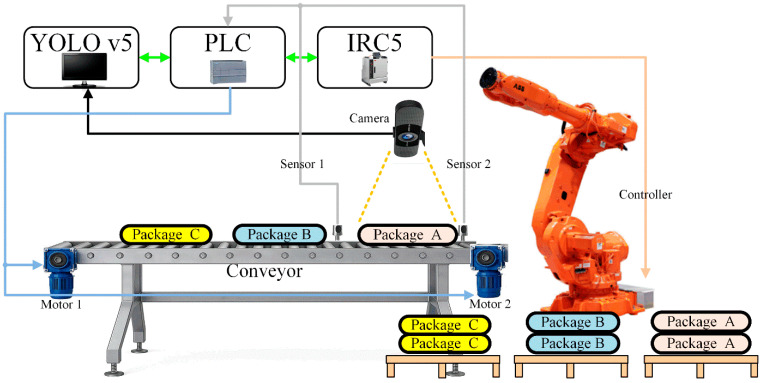
System architecture.

The stacking system developed in this study, supported by image processing, operates with a high level of synchronization among various industrial components and offers a fully integrated automation infrastructure. The system architecture is presented in Figure 1.

At the core of the system is the ABB IRB6640-130/3.2 industrial robotic arm, which is effectively used in applications requiring heavy load handling and a wide range of motion, owing to its 130 kg payload capacity and 3.2-m reach. The movements of the six-degree-of-freedom robotic arm are controlled by the IRC5 control unit, enabling the safe execution of complex stacking tasks. The programming process was carried out using ABB’s proprietary RAPID programming language, while offline testing and process optimizations were performed using RobotStudio simulation software (v2025.3).

The robot’s task initiation process begins with the analysis of images captured by a Basler acA2500-14uc industrial camera as the products move along the conveyor belt in real-time. This camera is paired with a Basler C125-0418-5M lens (Basler AG, Ahrensburg, Germany) (f = 4 mm, f/1.8 aperture) and provides a native resolution of 2592 × 1944 pixels using a 1/2.5″ CMOS sensor. It operates via a USB 3.0 interface at up to 14 fps. The selected lens configuration achieves an approximate 70° horizontal field of view at a 750 mm working distance, ensuring complete visual coverage of the conveyor belt area.

For dataset collection and offline model development, images were independently captured using an Apple iPhone 12 Pro Max(Apple Authorized Reseller, Konya, Türkiye) wide-angle camera module. This camera features a 12-megapixel resolution with a 26 mm focal length, f/1.6 aperture, and an image size of 3024 × 4032 pixels. Metadata from sample dataset images indicates typical capture settings including ISO 32 and a shutter speed around 1/1353 s, enabling sufficient illumination and detail under the laboratory-controlled lighting conditions.

Illuminance on the conveyor surface was kept within 480–520 lux, verified using a digital lux meter, and lighting was delivered by 4500 K neutral-white LED fixtures positioned symmetrically above the conveyor to prevent harsh reflections and shadows. To minimize temporal illumination variability, the LED lighting panels used in the setup operate at 25 kHz PWM frequency to eliminate perceptible flicker during image capture. The camera and lens assembly were allowed a 10-min warm-up period before data acquisition to stabilize sensor temperature and prevent thermal drift. Additionally, a periodic camera calibration protocol was applied on a weekly basis, involving white-balance and exposure alignment using a standardized gray reference card positioned on the conveyor surface. This procedure ensured consistent color fidelity and illumination uniformity across data collection and real-time operation, strengthening the reproducibility of the visual acquisition environment.

Image processing is carried out using custom software developed in Python, employing the YOLOv5 algorithm. This software evaluates visual features of the products such as color, text, or patterns to assign each item to the appropriate class, forming the core decision-making mechanism of the system. The resulting class ID from the image processing stage is transmitted to the central control unit of the system, a Siemens S7-1200 series (6ES7212-1HE40-0XB0) compact PLC.

The PLC receives data from the field through digital input/output modules and establishes bidirectional communication with the robotic arm via the Profinet communication protocol. The IRC5 control unit is integrated into the TIA Portal environment using a GSDML configuration file and is configured as a Profinet IO device. In this way, the PLC processes the received class information and triggers the corresponding stacking program for the robotic arm.

The robotic stacking operation was performed using an ABB IRB6640-130/3.2 manipulator and an IRC5 controller. Smooth motion was achieved by applying an S-curve velocity profile (*v_max_* = 1.2 m/s, *a_max_* = 2.8 m/s^2^, *j_max_* = 8.5 m/s^3^), resulting in continuous acceleration transitions and reduced mechanical vibration. The total cycle time per package was approximately 10 s, with 9.4 s allocated to robotic motion. However, because image processing and PLC communication are executed synchronously during the stacking motion, the system maintains an effective throughput of 6 packages per minute. Motion duration was further reduced using a trajectory blending algorithm implemented in ABB RAPID, which enabled smooth zone transitions (z20–z100) (see Table 1) and shortened the motion time by about 8% while maintaining ±0.2 mm positioning accuracy.

In the system, product transportation is carried out by a conveyor system driven by two three-phase asynchronous motors. One motor at the entry point and the other at the exit. These motors are controlled via contactors connected to the PLC outputs, enabling synchronized and safe system operation. To precisely determine the positions of products on the conveyor line, two optical sensors connected to the PLC’s digital inputs are used.

The first sensor (Sensor 1) detects when a product reaches the image processing area and sends a trigger signal to the PLC, initiating the classification process. The camera image is instantly transmitted to the YOLOv5 model, which determines the product’s class. As the product continues moving and reaches the second sensor (Sensor 2), the conveyor stops, and the classification is completed. The PLC then sends the stacking command corresponding to the class ID to the robot. The robotic arm executes the relevant code and places the product in its predefined stacking area (see Figure 1). This process is automatically repeated for each product.

The conveyor–sensor system was configured with a belt speed of 0.20 m/s, and the distance between Sensor 1 (image trigger) and Sensor 2 (stop signal for robotic picking) was 1100 mm. The camera was mounted 750 mm above the conveyor surface. With these parameters, a bag travels between the two sensors in approximately 5.5 s, which defines the available window for image capture and classification. The YOLOv5s-based image processing took 18 ms per frame, and PLC-to-robot command transmission via Profinet introduced a delay of approximately 0.20 s, both of which are negligible compared to the total cycle. The classification and robotic operations run synchronously; while the robot performs the stacking of one bag, the next bag is already captured and classified, enabling continuous synchronized operation without idle time.

The illuminance at the conveyor surface was increased from approximately 310 lux to a stable level of 500 ± 20 lux using 4500 K neutral-white LED lighting, reducing shadowing effects and glare. The camera was mounted 750 mm above the conveyor with a downward inclination of 15°, providing an approximate 70° horizontal field of view that fully covers the region of interest.

In addition, a fixed alignment cylinder was introduced to improve the positional stability of passing bags, maintaining a consistent lateral alignment with a clearance tolerance of approximately ±2 mm based on bag thickness. The conveyor belt operated at a constant 0.20 m/s, ensuring uniform motion and synchronized image capture.

These adjustments led to visibly enhanced edge contrast and overall image clarity, contributing to an approximate 2.5% increase in classification accuracy under identical test conditions. This optimized configuration ensures reliable spatial coordination between the imaging system and robotic manipulation components.

The system is configured to allow the simultaneous transport of three different product types on the conveyor. A total of 13 different product types are produced within the facility, processed across three separate filling stations. Each filling station supplies a distinct product type, and these are transferred onto a shared conveyor line. As the three different products move along the conveyor, the image processing system classifies them and directs them to their corresponding stacking areas.

Once the stacking process for a particular product group is completed, new products from the remaining 13 groups are loaded into the filling stations, allowing the system to continue operating with different product types. Owing to this cyclical structure, the system can sequentially process all product types, completing each cycle with predefined classification and stacking algorithms.

All hardware components used in the system were selected in accordance with industrial standards, with the aim of ensuring stable, continuous, and high-speed operation. This fully integrated structure, combining the PLC, conveyor, camera, robotic arm, and image processing algorithm, offers a reliable and flexible automation solution with minimal human intervention. Tested under real production line conditions, it presents a ready-to-deploy system architecture.

### 3.2. Dataset

The dataset used in this study was created from images manually captured in a real industrial environment. It consists of 13 classes, each representing a different type of industrial package. For each class, 50 images were collected, resulting in a total of 650 images. The images were manually taken using a mobile camera, with varying environmental factors such as lighting conditions, camera angles, surface reflections, and brightness levels taken into consideration during the capture process. This diversity was intended to improve the model’s robustness against variations that may be encountered in real-world applications. Additionally, to enhance the natural diversity of the collected raw images and better reflect the actual application environment, data augmentation techniques listed in Table 2 were applied.

**Figure 2:**
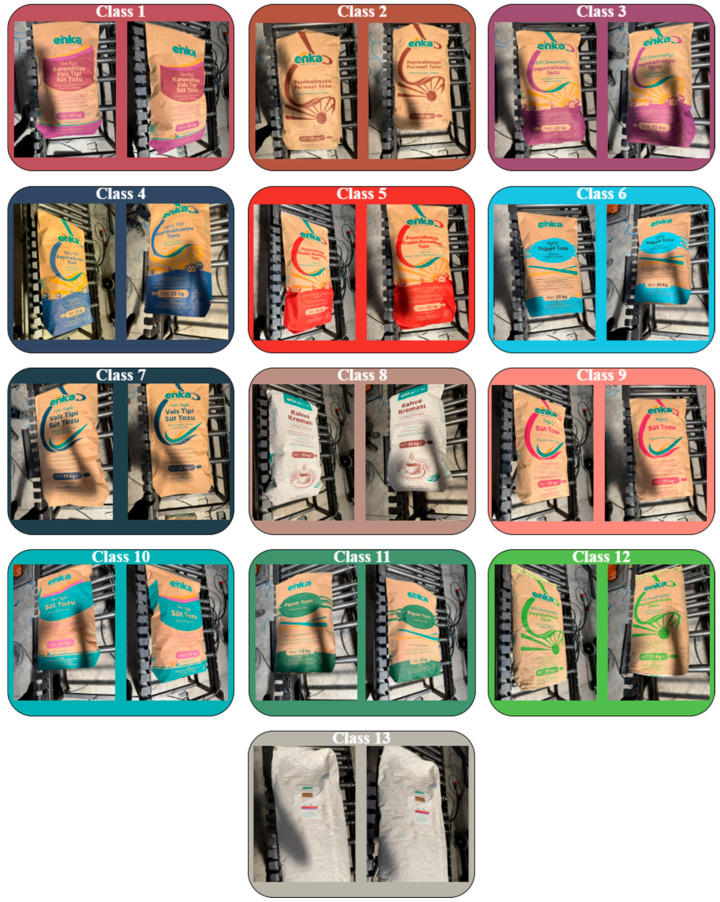
Sample images in the dataset.

This diversification enhanced the model’s sensitivity to rotation, orientation changes, lighting intensity variations, noise, and geometric distortions, thereby enabling high-accuracy classification, particularly in real-time industrial applications. Following data augmentation, the number of images in each class increased fivefold, resulting in a total of 3250 images across 13 classes. Sample images from the dataset are presented in Figure 2.

The labeling process was carried out manually using the open-source software LabelImg (v1.8.6). For each image, object classes were precisely defined using bounding boxes. All data was completely annotated, and the dataset contains no missing or corrupted samples.

Two data-splitting strategies were employed. In the first approach, the dataset was partitioned into 80% for training and 20% for validation. The second approach used a three-way split, dividing the data into 70% for training, 15% for validation, and 15% for testing.

Firstly, the dataset was divided into training (80%) and validation (20%) subsets for model training and evaluation.

Then the dataset was randomly divided into three subsets using a stratified 70-15-15 partition for training, validation, and testing, respectively. The test set remained completely unseen during model development to ensure unbiased performance evaluation.

Each of the 13 industrial bag classes differs slightly in dominant color tone, printing style, and material appearance. The majority are kraft-paper packaging, whereas Class 8 and Class 13 exhibit more reflective surfaces. These visual differences (as summarized in Table 3) define the discriminative challenge for the model, especially for low-contrast categories such as Class 8. All bags share similar geometric dimensions (500 × 900 × 140 mm), resulting in a consistent spatial distribution within the camera field of view.

### 3.3. YOLOv5

In this study, multiple YOLOv5 (You Only Look Once version 5) variants were trained and evaluated, including v5n, v5s, v5m, v5l, and v5x architectures with both Adam and SGD optimizers at 640 resolutions. Among these, the YOLOv5s (small) model was selected for real-time deployment due to its balance of speed and accuracy.

The YOLOv5 architecture family presents a scalable ladder of performance and computational demand across its variants: v5n, v5s, v5m, v5l, and v5x. YOLOv5n is the efficiency-focused version, featuring approximately 7.7 M parameters, achieving extremely fast inference with minimal hardware requirements, though this compact structure typically yields lower detection accuracy. YOLOv5s increases the size to around 9.1 M parameters, enhancing accuracy while still preserving real-time capability, making it a strong trade-off between speed and precision. YOLOv5m further expands to about 25.1 M parameters, improving detection performance at the cost of increased latency and computational load. YOLOv5l jumps to approximately 53.2 M parameters, designed for higher precision when resources permit and speed is less critical. At the peak of the spectrum, YOLOv5x features about 97.2 M parameters, delivering the highest detection performance among the family but demanding substantial computing power, which may limit its suitability for real-time deployment on standard hardware. Collectively, this range enables model selection tailored to the desired balance among speed, accuracy, and hardware resources.

**Figure 3:**
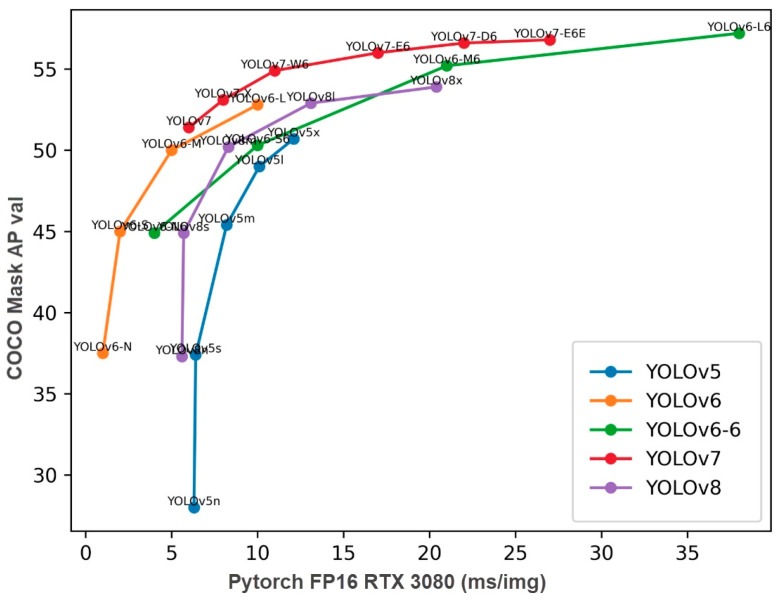
Comparison of accuracy (mAP50:95) and latency performance of different YOLO versions on the COCO dataset.

YOLOv5 is an open-source object detection algorithm based on PyTorch (v2.7.1) and is widely preferred in industrial applications due to its speed and lightweight nature during both training and inference processes [22,23,24]. A comparison of different YOLO versions in terms of accuracy (mAP50:95) and latency performance on the COCO dataset (in a T4 TensorRT10 FP16 environment) is presented in Figure 3 [25].

**Figure 4:**
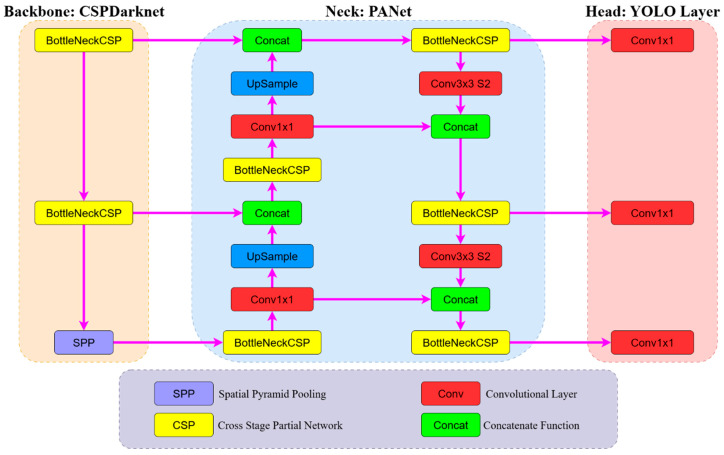
YOLOv5 architecture.

As shown in Figure 3, the YOLOv5s model indicated by the “5s” label on the graph draws attention by achieving approximately 46% mAP50:95 accuracy, despite operating with an exceptionally low latency of around 2 ms. This makes YOLOv5s a highly suitable choice for real-time applications. Particularly in embedded systems with limited computational resources or industrial applications with low hardware requirements, YOLOv5s offers an optimal balance between speed and accuracy. Although next-generation YOLO models (YOLOv8–YOLOv11) deliver higher accuracy, they are observed to be more costly in terms of processing latency [26]. Furthermore, the modular, flexible, and efficient structure of the YOLO architecture compared to other versions makes the lightweight YOLOv5s model well-suited for this study. The architecture of YOLOv5 is presented in Figure 4.

YOLOv5s uses the CSPDarknet architecture as its backbone, the Path Aggregation Network (PANet) structure in the neck, and an anchor-based structure in the head. This configuration enables effective object detection from both low- and high-resolution feature maps. The model performs both classification of objects and regression-based prediction of their location (bounding boxes) within each image [27,28,29,30,31]. The technical specifications and performance metrics of the YOLOv5 model used in this study are presented in Table 4.

To validate the selection of the optimization algorithm, the model was trained using both SGD and Adam under identical hyperparameters.

### 3.4. Performance Metrics

The performance of the YOLOv5s model during training and testing was evaluated using various metrics commonly used in the field of computer vision. These metrics comprehensively reflect the model’s accuracy, sensitivity, reliability capability, and spatial precision in object detection.

#### 3.4.1. Confusion Matrix

**Figure 5:**
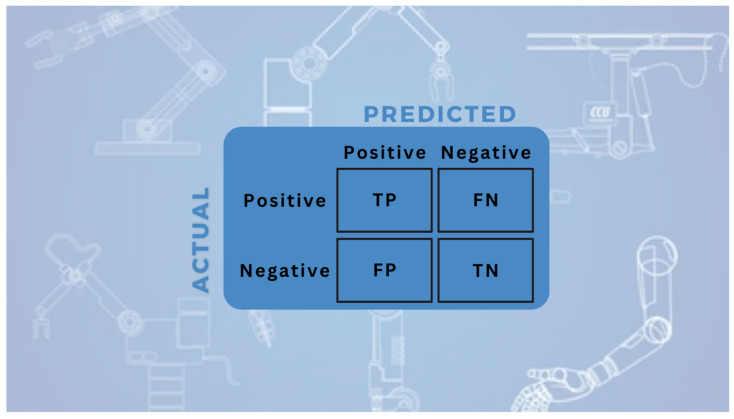
Two-class confusion matrix.

**Figure 6:**
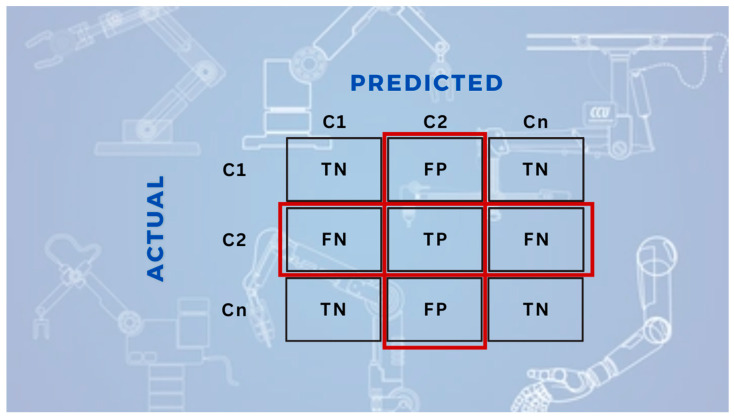
Multi-class confusion matrix.

In multi-class object recognition tasks, the fundamental structure used to summarize whether the model’s predictions for each class are correct or incorrect is the confusion matrix. Each cell numerically represents the relationship between the actual and predicted classes [32,33]. The two-class confusion matrix representation is given in Figure 5, and the multi-class confusion matrix is presented in Figure 6.

True Positive (TP) indicates the number of instances correctly classified as positive by the model. False Positive (FP) represents the number of instances incorrectly classified as positive. False Negative (FN) refers to instances that were incorrectly classified as negative, while True Negative (TN) represents the number of instances correctly classified as negative. These four fundamental components serve as the basis for both class-specific and overall model evaluation [34,35,36,37].

#### 3.4.2. Precision

Precision indicates how many of the model’s positive predictions are actually correct. A high-precision value means the model rarely detects a non-existent object as if it were present. The calculation formula is given in Equation (1) [38,39].

(1) $$Precision=\frac{\sum {TP}_{i}}{\sum \left({TP}_{i}+{FP}_{i}\right)}$$

#### 3.4.3. Recall

Recall indicates how many of the actual positive instances the model was able to correctly identify. A high recall value suggests that the model has a low miss rate. The calculation formula is provided in Equation (2) [38,39].

(2) $$Recall=\frac{\sum {TP}_{i}}{\sum \left({TP}_{i}+{FN}_{i}\right)}$$

#### 3.4.4. Intersection over Union (IoU)

In object detection models, IoU measures how much the predicted bounding box overlaps with the ground truth box. The IoU value ranges between 0 and 1, with predictions having an IoU of 0.5 or higher typically considered as “correct detections.” The calculation formula is given in Equation (3) [40].

(3) $$IoU=\frac{Prediction\;Box\cap Actual\;Box}{Prediction\;Box\cup Actual\;Box}$$

#### 3.4.5. Mean Average Precision (mAP)

mAP@0.5 is the mean of the average precision values calculated for each class, where the IoU threshold is fixed at 0.5 [41,42]. The calculation formula is given in Equation (4).

(4) $$mAP@50=\frac{1}{N}\sum_{i=1}^{N}{AP}_{i}^{IoU=0.5}$$

mAP@0.5:0.95 is the mean average precision score calculated over 10 different IoU threshold values ranging from 0.5 to 0.95 in increments of 0.05. This metric provides a much more precise evaluation of the model’s performance in terms of both correct classification and accurate localization [41,42]. The calculation formula is provided in Equation (5).

(5) $$mAP@0.5:0.95=\frac{1}{10N}\sum_{j=0}^{9}\sum_{i=1}^{N}{AP}_{i}^{IoU=0.5+0.05j}$$

#### 3.4.6. Box Loss (Bounding Box Regression Loss)

It measures the spatial difference between the predicted box and the ground truth box. The YOLOv5 Complete IoU (CIoU) loss function is used as the basis. In the YOLOv5s model, the advanced loss function used to measure the difference between the predicted and ground truth boxes is defined by CIoU as shown in Equation (6) [43]:

(6) $$L_{CIoU}=\;1\;-\;IoU\;+\;\frac{\left(\rho^{2}(b,\;b^{gt}\right)}{\;c^{2}}\;+\;\alpha v$$

Here:

*b*: predicted bounding box.

*b^gt^*: center of the ground truth bounding box.

*ρ*: Euclidean distance between the centers of the two boxes.

*c*: diagonal length of the smallest box that encloses both the ground truth and the predicted bounding boxes.

*v*: aspect ratio difference between the boxes.

*α*: weighting factor (adjusted based on IoU).

This loss function produces more precise results by considering not only the overlap ratio but also the positional and shape similarities between the boxes [43].

#### 3.4.7. Objectness Loss

This is a loss function used to predict whether a real object exists within a grid cell or anchor box. The model generates an “objectness score” for each anchor box, which is a probability ranging between 0 and 1.

In the YOLO architecture, each grid cell is expected to be responsible for only one object. If there is no object, the model should produce a low objectness value (≈0) for that anchor; if there is an object, the value should be high (≈1). This metric helps reduce false positives by preventing incorrect object predictions in background regions. Minimizing the objectness loss is especially important in images with complex backgrounds, as it significantly improves the overall accuracy of the model [44]. The calculation formula is given in Equation (7).

(7) $$L_{obj}=\frac{1}{N}\sum_{i=1}^{N}[{-y}_{i}\log\left({\hat{y}}_{i}\right)-\left(1-y_{i}\right)log(1-{\hat{y}}_{i})]$$

Here:

$y_{i}$: ground truth (0 or 1; indicates whether there is an object in the anchor box).

${\hat{y}}_{i}$: objectness score predicted by the model.

*N*: number of anchors.

#### 3.4.8. Classification Loss

Classification loss is a key metric that measures the model’s ability to correctly predict the class of an object within the detected regions. In the YOLOv5 architecture, each anchor box produces multi-class predictions using a sigmoid activation function. Therefore, independent predictions are made for each class, and the contribution of each class is calculated separately. This structure increases flexibility in cases of class imbalance and enables more precise classification in scenes containing multiple objects [44]. The calculation formula is provided in Equation (8).

(8) $$L_{cls}=\frac{1}{M}\sum_{j=1}^{M}\sum_{k=1}^{C}[{-y}_{jk}\log\left({\hat{y}}_{jk}\right)-\left(1-y_{jk}\right)log(1-{\hat{y}}_{jk})]$$

Here:

$y_{jk}$: ground truth value for class k (0 or 1; for anchor j).

${\hat{y}}_{jk}$: class probability predicted by the model.

*M*: number of anchors containing objects.

*C*: number of classes.

### 3.5. Model Configuration and Training Process

In this study, YOLOv5 variants, an open-source and lightweight model, were used for object detection. The model was initialized with pre-trained weights and fine-tuned using the dataset described above. The training process was conducted on a high-performance computing system equipped with an NVIDIA GeForce RTX 4060 Ti (8 GB) GPU, an Intel Core i7-12700 K @ 3.61 GHz CPU, and 64 GB (4800 MHz) of RAM.

During training, input images were processed at a resolution of 640 × 640 pixels. The training was limited to 100 epochs, with an early stopping mechanism (patience = 50) activated to prevent unnecessary extension of the learning process. The mini-batch size was adjusted according to the computational demands of each YOLOv5 variant to ensure stable training and full compatibility with available GPU memory. The lightweight models (YOLOv5n and YOLOv5s) were trained using a batch size of 16, while the medium variant (YOLOv5m) also maintained a batch size of 16 due to moderate memory requirements. The larger YOLOv5l architecture necessitated reducing the batch size to 8, and the most computationally intensive model, YOLOv5x, was trained with a batch size of 4 to prevent memory overflow during training. This adaptive configuration balanced training efficiency with hardware limitations across all model variants. The model was trained using a transfer learning approach. Key performance metrics such as mAP@0.5, precision, recall, and F1-score were continuously monitored throughout the training. Additionally, Box Loss, Objectness Loss, and Classification Loss values were evaluated to track training progress. This configuration aimed to enhance overall model accuracy while minimizing the risk of overfitting.

## 4. Results

In this section, the training, validation, and test performances of the YOLOv5-based image processing model are presented comprehensively through quantitative metrics, visual comparisons, and class-based analyses. The model’s ability to distinguish between 13 different classes of packages is evaluated using loss metrics recorded during training, performance curves, class-wise accuracy rates, and prediction examples.

**Figure 7:**
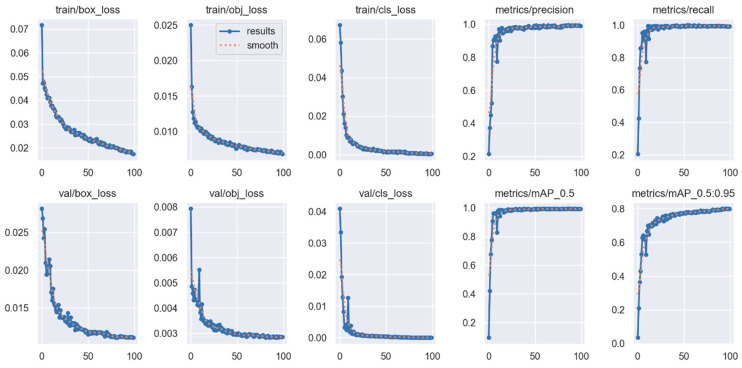
Training and validation metrics of YOLOv5s (80-20 split, SGD).

The YOLOv5s model was trained using an 80-20 training-validation data split for a total of 100 epochs with the SGD optimizer. Performance metrics demonstrated consistent improvement throughout training. Although an early-stopping patience of 50 was defined, the stopping criterion was never activated, since the validation loss did not exhibit degradation and continued to show incremental improvements across the epochs. The evolution of loss values and performance metrics for both training and validation phases is illustrated in Figure 7.

As shown in Figure 7, the values for box loss, objectness loss, and classification loss monitored during both the training and validation phases consistently decreased as the number of epochs increased. In the train/box_loss and val/box_loss graphs, it is observed that errors related to bounding box localization steadily declined throughout the training process, and this trend was maintained in the validation set as well. Similarly, train/obj_loss and val/obj_loss values dropped rapidly, reaching approximately 0.003, indicating the model’s high accuracy in distinguishing between background and objects.

When evaluating train/cls_loss and val/cls_loss, a significant improvement in classification errors is observed; the loss decreased markedly in the early stages of training and approached near-zero values in later epochs. This demonstrates that the model developed a high level of sensitivity in distinguishing between class labels.

In terms of model performance, both SGD and Adam optimizers were evaluated using epoch-wise tracking of precision, recall, and mAP indicators. As shown in Table 5, the YOLOv5s model trained with SGD achieved rapid performance gains within the first five epochs, where precision, recall, and mAP@0.5 increased from 0.214 → 0.903, 0.205 → 0.952, and 0.094 → 0.962, respectively. This steep learning curve indicates that the model effectively captured discriminative features from the dataset at an early stage.

After the 10th epoch, performance continued to improve at a slower rate, eventually stabilizing around precision ≈ 0.99, recall ≈ 0.99, and mAP@0.5 ≈ 0.994. The more challenging mAP@0.5:0.95 metric also showed continuous progress, reaching approximately 0.80 by the end of training. These results confirm that the model not only localized accurately but also maintained robust performance across stricter IoU thresholds.

Similarly, when trained using Adam (see Table 6), the model showed competitive performance with mAP@0.5 ≈ 0.985 and mAP@0.5:0.95 ≈ 0.746 at final epochs. Although Adam achieved quicker early convergence, slight fluctuations in the validation curves were observed near the end of training, leading to slightly lower final accuracy compared to the SGD configuration.

Similarly, when trained with the AdaBoB optimizer (see Table 7), the model exhibited rapid and stable learning behavior throughout the training process. Although the initial epochs began with relatively low precision (0.166) and mAP@0.5 (0.159), performance improved sharply within the first five epochs, reaching 0.732 precision, 0.822 recall, and an mAP@0.5 of 0.862. By the 10th epoch, mAP@0.5 had already surpassed 0.96, indicating fast convergence comparable to the Adam configuration. From the 20th epoch onward, the performance curves stabilized at high levels, with mAP@0.5 values consistently above 0.99 and mAP@0.5:0.95 showing steady gains, peaking at approximately 0.781 near the final epoch.

As seen in Table 5, the epoch-based performance metrics of the YOLOv5s model trained with SGD demonstrate rapid and steady learning throughout the training process. Although precision (0.214), recall (0.205), and mAP@0.5 (0.094) were initially low at the first epoch, the model quickly improved, reaching 0.903 precision, 0.952 recall, and 0.962 mAP@0.5 by the 5th epoch. By the 10th epoch, mAP@0.5 had already risen to 0.981, after which the performance curves gradually stabilized. From approximately the 30th epoch onwards, both precision and recall consistently exceeded 0.98, and mAP@0.5 remained close to 0.99. The more rigorous mAP@0.5:0.95 metric continued improving during the later epochs, reaching its peak value of 0.799 at epoch 98, demonstrating strong generalization capability across varying IoU thresholds.

As presented in Table 6, the Adam optimizer also resulted in strong convergence, achieving mAP@0.5 ≈ 0.985 and mAP@0.5:0.95 ≈ 0.746 in the final epochs. Adam displayed faster initial convergence than SGD; however, slight fluctuations in the validation trend were observed during later stages of training, and the final accuracy remained marginally below that of the SGD configuration, indicating comparatively reduced stability near convergence.

Table 7 further shows that the AdaBoB optimizer achieved competitive performance, reaching mAP@0.5 values above 0.99 by epoch 20 and maintaining high performance throughout the remaining training period. AdaBoB exhibited faster mid-stage convergence and more stable late-stage performance than Adam while achieving final accuracy levels comparable to SGD. These results collectively demonstrate that, while all three optimizers are capable of effective training, SGD provided the most stable and consistent overall performance for this application, justifying its selection for real-time system deployment.

The results indicate that the model maintained high performance over an extended period without falling into overfitting. This balanced success throughout training was supported by the quality of the dataset, the applied data augmentation strategies, and the use of well-tuned hyperparameters.

**Figure 8:**
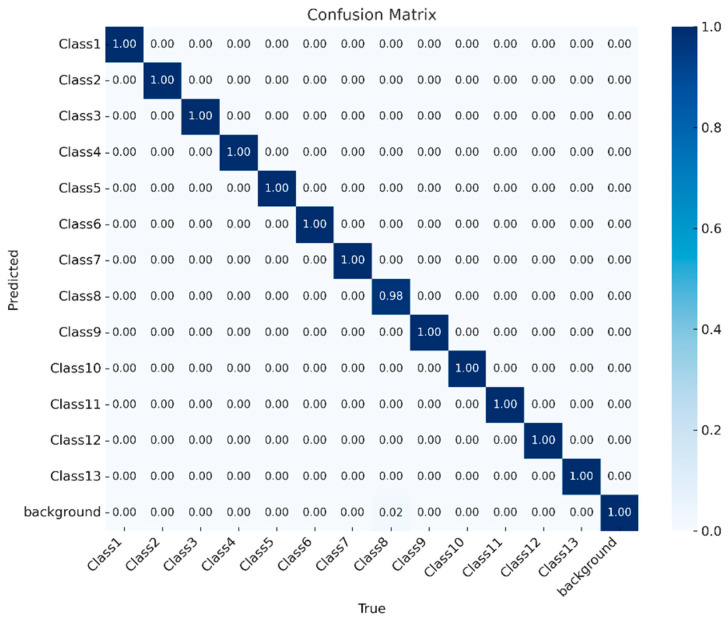
Confusion matrix of YOLOv5s (80-20 split, SGD).

Among the three optimizers evaluated (SGD, Adam, and AdaBoB), the SGD configuration yielded the most stable and highest overall performance. Therefore, the confusion matrix of this best-performing model is shown in Figure 8, with detailed class-wise performance metrics provided in Table 8.

After evaluating the evaluations presented in Figure 8 and Table 8 regarding the model’s class-wise performance, the confusion matrix visually demonstrates the prediction accuracy for each class. The fact that the diagonal values are close to 1.00 indicates that there was virtually no confusion between classes. While the correct classification rate was nearly 100% for all classes, only Class 8 showed a slightly lower accuracy at 98% (0.98), with a small portion (2%) of background data mistakenly classified as Class 8. Apart from this minor error, the model demonstrated strong discriminative capability in distinguishing both foreground and background.

Table 8 presents the precision, recall, mAP@0.5, and mAP@0.5:0.95 values calculated individually for each class. With precision and recall scores ranging between 0.99 and 1.00 for most classes, the results show that the model performs highly accurate predictions with minimal false positives or false negatives. Notably, Class10 achieved the highest precision at 0.997. The lowest precision value was observed in Class8 (0.947), which aligns with the minor misclassification noted in the confusion matrix. While mAP@0.5 remained steady at 0.995 across all classes, mAP@0.5:0.95 values showed slight variations between classes, indicating that localization accuracy may vary slightly depending on the class. The highest mAP@0.5:0.95 was recorded for Class9 (0.875) and the lowest for Class8 (0.686).

**Figure 9:**
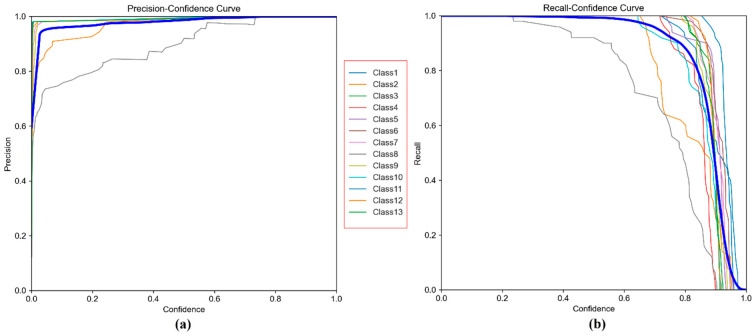
Curves showing the relationship between the model’s precision (**a**) and recall performance (**b**) and confidence scores.

These findings suggest that, although the model demonstrates high overall performance, there is still room for improvement in a few class-specific areas. The curves showing the relationship between precision and recall, and confidence scores are presented in Figure 9.

The Precision–Confidence curve (Figure 9a) shows that the model maintains near-perfect precision even at high confidence levels. For most classes, precision curves quickly reach the 1.00 level early on and sustain this value as confidence increases. This indicates that predictions made with high confidence are largely correct, meaning the model has a low false positive rate. On average, the precision curve across all classes reaches 100% at a confidence level of 0.879, suggesting that the system can operate reliably with safe threshold values in industrial applications.

The Recall–Confidence curve (Figure 9b) illustrates the model’s sensitivity and how increasing the confidence threshold affects the number of detected samples. As the curves show, recall gradually decreases as confidence rises. This suggests that, at higher confidence levels, the model makes fewer predictions, causing some correct instances to be missed, recall drops. Notably, this decline starts earlier for certain classes like Class 8 and Class 12, indicating that the model is more cautious with uncertain predictions in those cases. However, the average across all classes shows that the model maintains high recall (close to 1.00) even at lower confidence levels.

**Figure 10:**
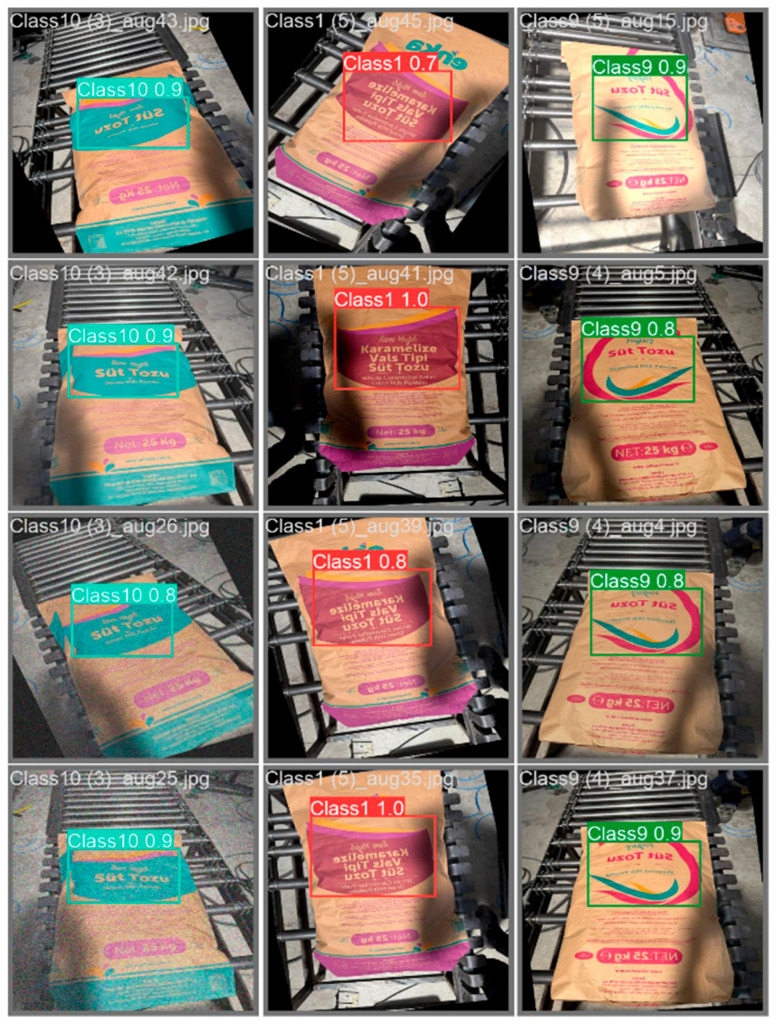
Sample class predictions made by YOLOv5s (80-20 split, SGD) model on the validation dataset.

Prediction examples from the validation dataset are presented in Figure 10. These images show that the model successfully classifies different types of packages with high accuracy and confidence scores. For example, predictions for Class 10, Class 9, and Class 1 mostly exceed a confidence score of 0.90. However, in some cases, lower confidence scores (around 0.70) are observed, indicating less certainty. Such cases are often caused by factors like the angle of the text on the package, shadows, or partially visible labels. Overall, the model’s strong performance on validation data supports its suitability for real-time industrial applications.

Table 9 presents the final test-set performance obtained using the SGD, AdaBoB, and Adam optimizers under the 80-20 split configuration. Among the three, the SGD optimizer achieved the strongest overall performance, yielding the highest mAP@0.5 (0.993) and mAP@0.5:0.95 (0.799), indicating superior generalization capability on unseen data. AdaBoB demonstrated highly competitive results, closely approaching the performance of SGD, particularly in recall, showing its effectiveness as a modern adaptive optimizer. In contrast, Adam recorded lower values across all four metrics, reflecting less stable convergence and reduced detection accuracy compared to SGD and AdaBoB for this dataset.

**Figure 11:**
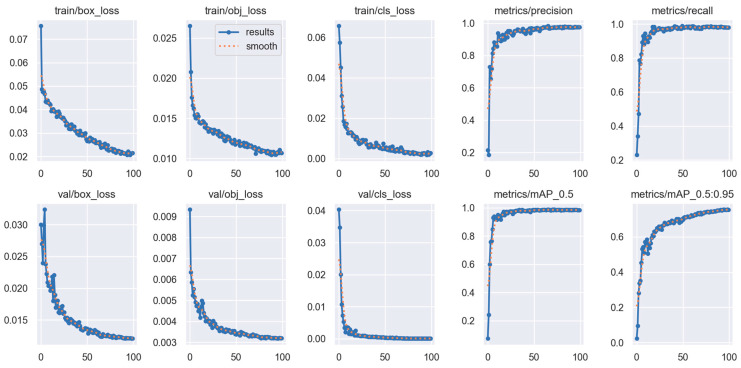
Training and validation metrics of YOLOv5s (70-15-15 split, SGD).

The YOLOv5s model was trained using a 70-15-15 split for training, validation, and testing over 100 epochs with the SGD optimizer. The progression of training and validation loss and performance metrics is illustrated in Figure 11.

The learning curves in Figure 11 demonstrate a stable and reliable training process. All loss components (box, objectness, and classification) show a smooth and consistent decline across epochs for both training and validation, indicating effective optimization without signs of overfitting. The validation and training curves remain closely aligned, confirming that the model performs well on unseen data. In parallel, performance metrics including precision, recall, mAP@0.5, and mAP@0.5:0.95 rise rapidly during the initial epochs and gradually stabilize at high values as training progresses. These outcomes show that the YOLOv5s model successfully converged while maintaining strong detection performance under the 70-15-15 data split with the SGD optimizer.

Table 10 demonstrates a notable improvement in detection performance as training progresses. At the initial epochs, the model exhibits low precision, recall, and mAP values due to limited learned feature representations. A sharp performance increase is observed by Epoch 5, where both precision (0.81456) and recall (0.82213) surpass the 0.80 threshold, accompanied by a marked rise in mAP@0.5 (0.84706). Continued optimization beyond Epoch 10 results in performance stabilization, with mAP@0.5 exceeding 0.97 by Epoch 20. Throughout later epochs, the metrics maintain consistently high levels, reflecting strong and reliable detection accuracy. By the final epochs (98–99), the model reaches its peak values with precision of approximately 0.975, recall of around 0.981, and mAP@0.5 surpassing 0.984, while mAP@0.5:0.95 approaches 0.754. These results indicate that the YOLOv5s model rapidly acquires essential object detection capabilities and sustains excellent performance through the remainder of training under the 70-15-15 split configuration.

In contrast, Table 11 presents the performance of the YOLOv5s model trained using the Adam optimizer under the same 70-15-15 split configuration. Although Adam achieved competitive performance, the progression exhibited greater fluctuations in the early and mid-training stages, particularly in recall and mAP values. After approximately 40 epochs, the metrics began to stabilize, reaching precision ≈ 0.95, recall ≈ 0.98, and mAP@0.5 ≈ 0.97 toward the final epochs. While the final results remain strong, the slight instability during training and slightly lower peak performance compared to the SGD configuration indicate that SGD offers a more reliable and robust optimization strategy for this industrial application.

Table 12 shows that the AdaBoB optimizer enabled rapid and stable convergence under the 70-15-15 split. The model reached strong performance by Epoch 10 (mAP@0.5 = 0.953) and continued improving steadily, peaking at Epoch 98 with precision of 0.966, recall of 0.986, and mAP@0.5 of 0.981. The mAP@0.5:0.95 value also increased consistently, reaching 0.728, indicating enhanced robustness across IoU thresholds. Overall, AdaBoB provided smooth late-stage gains and stable learning behavior.

As shown in Table 10, the YOLOv5s model trained with the 70-15-15 split using the SGD optimizer exhibited rapid and stable convergence. Although the initial epoch values were low (Precision = 0.214, Recall = 0.230, mAP@0.5 = 0.074), performance improved sharply within the first 5 epochs, with mAP@0.5 reaching 0.847. By the 20th epoch, mAP@0.5 exceeded 0.97, and from the 30th epoch onwards, Precision and Recall consistently remained above 0.95, with mAP@0.5 stabilizing around 0.98–0.99. The mAP@0.5:0.95 metric continued to increase gradually, reaching a peak of approximately 0.754 at epoch 98, indicating strong performance reliability even under stricter IoU thresholds.

When Adam was used for training (Table 11), the model also achieved strong performance, but with greater volatility, especially in the early epochs. Although Adam showed faster early learning at specific checkpoints, final mAP values (mAP@0.5 = 0.975 and mAP@0.5:0.95 = 0.694 at epoch 99) remained slightly lower than those obtained with SGD. These fluctuations suggest that Adam provided quicker initial convergence but lacked the late-stage stability observed in the SGD configuration.

AdaBoB-based training (Table 12) demonstrated competitive performance, achieving mAP@0.5 = 0.981 and mAP@0.5:0.95 = 0.728 at epoch 98. While AdaBoB surpassed Adam in final mAP scores and achieved smoother mid-to-late training progression, SGD still provided the most stable and highest overall performance across all key metrics under the 70-15-15 split. Therefore, the SGD configuration was selected for reporting confusion matrix and class-wise results, as it demonstrated the best balance of accuracy and training stability for real-time deployment.

For the 70-15-15 split, the SGD configuration achieved the highest and most stable performance, reaching a final mAP@0.5 of 0.9843 and mAP@0.5:0.95 of 0.754, outperforming both AdaBoB and Adam, which showed slightly lower accuracy and greater fluctuation during later epochs.

**Figure 12:**
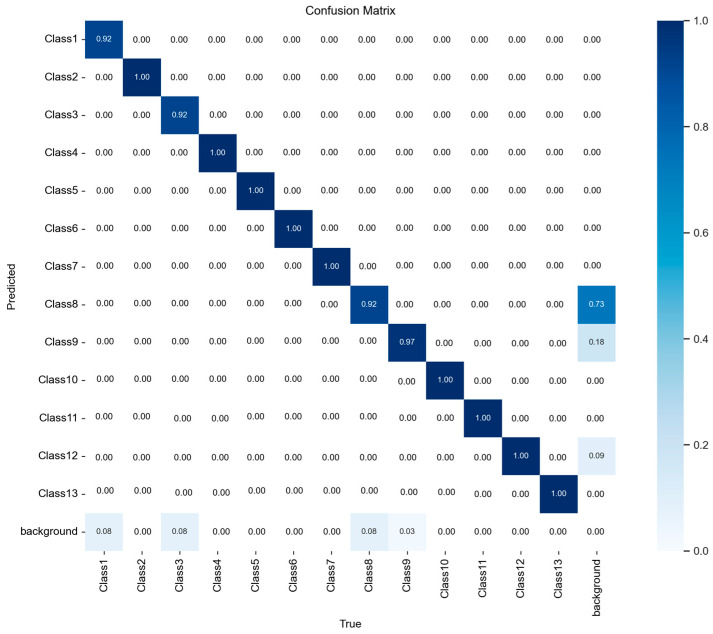
Confusion matrix of YOLOv5s (70-15-15 split, SGD).

The confusion matrix in Figure 12 shows that the YOLOv5s model achieved excellent classification performance across all 13 classes and background. Most classes exhibit perfect prediction accuracy, with values of 1.00 on the diagonal, indicating that the model consistently distinguishes these categories without errors. Minor misclassifications appear primarily between Class 8, Class 9, and the background, with small proportions such as 0.56 and 0.12 where background samples were occasionally predicted as target classes, and 0.05–0.03 where the reverse occurred. Overall, the confusion matrix highlights strong model reliability, demonstrating that YOLOv5s provides highly accurate detection and classification under the 70-15-15 split using the SGD optimizer.

As shown in Table 13, class-wise performance analysis confirms that the proposed model demonstrates strong and reliable detection capability across all classes. Precision and recall values mostly remain above 0.98, indicating that both false-positive and false-negative predictions are minimal for the majority of classes. The highest precision value was achieved in Class 4 and Class 10 (0.997), while the lowest occurred in Class 8 (0.800), which aligns with its relatively low text contrast and semi-glossy surface characteristics noted earlier.

Similarly, recall values also show high consistency, with 11 out of 13 classes reaching 1.00, confirming that nearly all relevant objects were correctly detected. Although Class 1 and Class 8 exhibited slightly lower recall scores (0.892 and 0.838), their accuracy remains acceptable within industrial tolerance limits.

Regarding localization performance, mAP@0.5 values remained high, ranging from 0.868 to 0.995 across classes. However, the more stringent metric mAP@0.5:0.95 reveals greater variability, particularly in visually challenging categories. The highest localization performance was observed in Class 12 (0.834), whereas Class 8 recorded the lowest score (0.644), indicating that precise boundary estimation becomes more difficult when visual features are less distinct.

**Figure 13:**
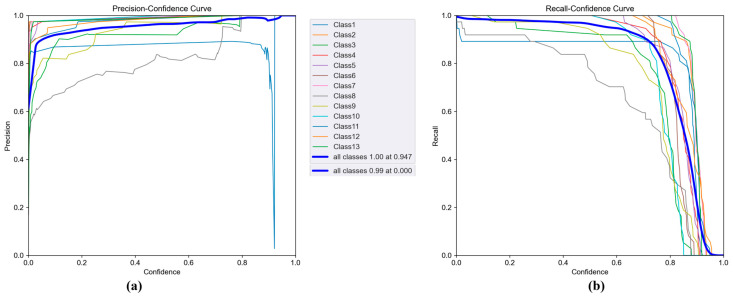
Curves showing the relationship between the YOLOv5s (70-15-15 split, SGD) model precision (**a**) and recall performance (**b**) and confidence scores.

**Figure 14:**
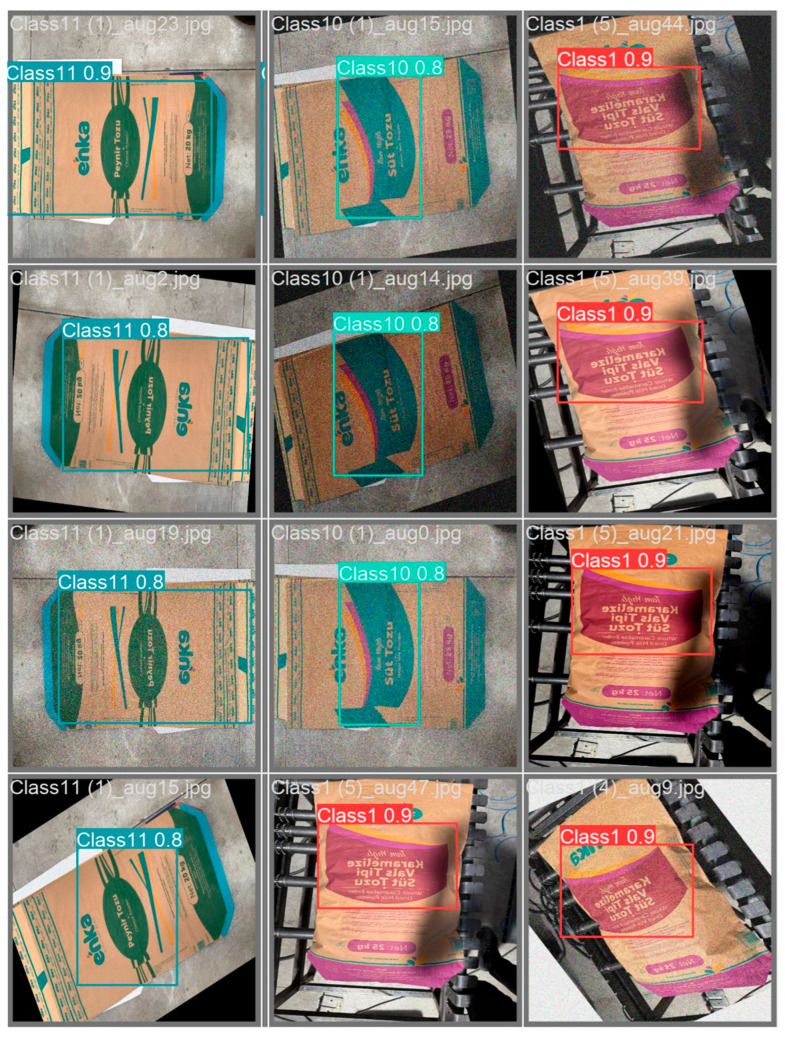
Sample class predictions made by the YOLOv5s (70-15-15 split, SGD) model on the test dataset.

Figure 13a shows consistently high precision across confidence thresholds, indicating very few false positives for most classes. The overall curve remains near 1.0, reflecting strong prediction accuracy. Figure 13b demonstrates that recall also stays high at lower thresholds and declines gradually as confidence increases, representing an expected balance between detection sensitivity and strictness. Overall, the curves confirm reliable object detection performance across all classes. Typical true positives and failure modes, based on sample detections by YOLOv5s (70–15–15 split, SGD) on the test set, are shown in Figure 14.

Table 14 compares the final test-set performance obtained using SGD, AdaBoB, and Adam. As shown, the SGD optimizer achieved the highest mAP@0.5 (0.974) and mAP@0.5:0.95 (0.741), indicating superior performance reliability on unseen data. AdaBoB delivered competitive accuracy, outperforming Adam and approaching SGD performance, while Adam recorded comparatively lower scores across all metrics.

### 4.1. Graphical User Interface (GUI) Design

**Figure 15:**
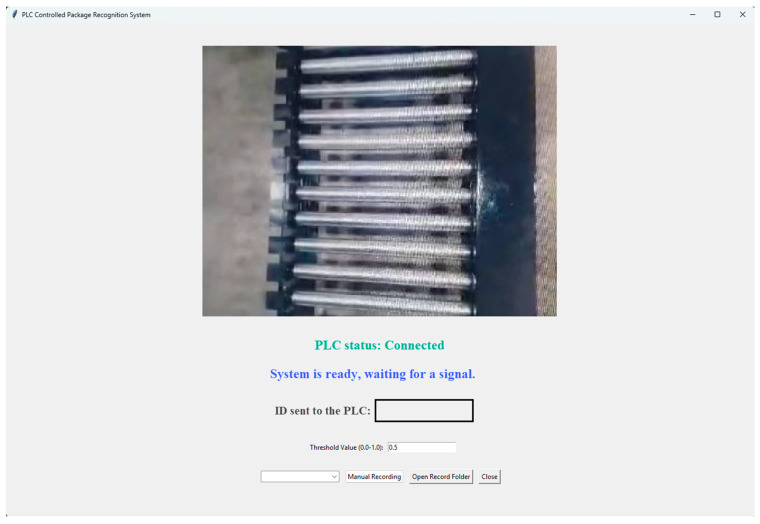
General view of the developed user interface.

In this study, the user interface design was developed using Python’s built-in GUI library, Tkinter. Real-time video streaming is provided through a USB camera connected to the system, and the captured frames are processed by the trained YOLOv5s-based model. The overall view of the developed user interface is shown in Figure 15.

The trained detector is deployed on an x86-64 Windows 7 industrial PC (Intel Core i5, integrated graphics) as a stand-alone GUI application. The system is packaged with PyInstaller and distributed via Inno Setup, bundling only the required YOLOv5 runtime, the model artifact (.pt), and PLC I/O dependencies. Inference runs on PyTorch (CPU, FP32) using YOLOv5 via DetectMultiBackend with batch size = 1 and 640 × 640 input resolution. The GUI communicates with a Siemens S7-1200 PLC through snap7, logs events to Excel via OpenPyXL, and provides an adjustable confidence threshold for operators. The deployed model file (best.pt) is ~14.2 MB; the single-file installer footprint is ~180 MB. Measured end-to-end latency (camera → inference → PLC write) on CPU FP32 is 74.3 ± 12.1 ms per frame (N = 200, 640 px, batch = 1), and peak resident memory (RSS) during inference is ~520 MB. To improve determinism and throughput on this hardware, we use a fixed input resolution, batch = 1 streaming, and retain FP32 on CPU (half precision is not used on CPU to preserve operator compatibility).

The “PLC status” indicator on the interface provides real-time feedback to the user regarding the connection status between the system and the industrial control unit (PLC), notifying whether the network connection is established or if there is a connection error. Following the classification process, the predicted class ID and the corresponding package name are automatically displayed under the “ID sent to the PLC” section and simultaneously transmitted to the PLC system. The system processes only those classification results that exceed a predefined confidence threshold. By default, this threshold is set to 0.5, but it can be adjusted by the user directly through the interface.

To handle undefined or uncertain scenarios, the system includes specially configured operator interaction and safety protocols. When a product is detected by Sensor 1 on the conveyor line and the image processing routine is initiated, the system allows a maximum of 5 s for classification. If no prediction is made within this timeframe, a warning is displayed on the GUI. To prevent production delays, the operator can manually enter the class ID using the “Manual Recording” module on the interface. If no manual entry is made and no new signal is received from Sensor 1, the robotic arm enters “standby mode” for safety, halting all movements. This structure helps avoid unplanned stoppages and prevents uncontrolled system operation, adding an extra layer of safety. Additionally, it helps prevent incorrect stacking of uncertain or unidentifiable items that could not be properly classified through machine vision.

All system records such as date, time, class ID, and whether the entry was manual or automatic are logged, supporting both operational traceability and quality control monitoring. Moreover, in cases of undefined classifications, the system can automatically archive the corresponding product image with a timestamp in a separate folder based on the operator’s manual entry. This allows uncertain or misclassified examples to be added to the labeled dataset for future model improvement.

### 4.2. Software System and Real-Time Performance of the Fully Integrated System

The image processing and robotic stacking system developed for use in the factory environment is capable of recognizing and directing a total of 13 different types of bags. The system identifies, classifies, and directs each incoming bag to the appropriate stacking point. Technically, it can handle all 13 product classes, but, due to daily production plans, only three different types of bags are processed each day. Therefore, instead of operating at full capacity, the system runs with a limited product set, aligned with the structure and scheduling of the production line. During field deployment, bags naturally arrived in a consistent lower-edge-first orientation but occasionally exhibited slight right-side tilting or small lateral shifts, representative of normal conveyor handling variability.

The YOLOv5-based classification model is integrated with the industrial conveyor system and analyzes each incoming bag in real time. The model’s average inference time is 18 milliseconds, which includes the time required to feed the image into the model and determine the class. When Sensor 1 (see Figure 1) detects the front part of a bag, the image processing software starts scanning. However, since it takes a few seconds for the entire bag to enter the camera’s field of view, the classification is typically completed within 3–4 s after the sensor is triggered.

Once classification is complete and the result is sent to the robot system via the PLC, the robotic arm moves to the target bag, grasps it, and places it on the correct pallet. This entire operation, which includes speed limitations, safety margins, motion smoothing (S-curve algorithms), and returning to the initial position, takes approximately 9–10 s. As a result, the full cycle time from sensor trigger through observation, classification, robot movement, and stacking is completed in about 10–11 s.

However, this cycle time does not reset for each individual bag. While the robot is performing the stacking task, image processing begins for the next bag. Owing to this synchronization, the system operates efficiently by overlapping process times. This structure allows the system to theoretically process 5–6 bags per minute.

Nevertheless, due to production planning requirements, this speed is deliberately limited to 4 bags per minute. Although the image processing infrastructure and robot system are capable of higher throughput, intentional wait times are defined to maintain synchronization with the production line and product flow. This not only ensures smooth integration with the current production tempo but also offers flexibility to scale up operations in the future if needed.

During real-time field tests, a dedicated test environment was first created for the YOLOv5s-based software system. Trials were conducted using 50 bags from each class, totaling 650 bags. After involving all 13 classes in the tests, the tested class information and results are presented in Table 15.

In the initial field test, 644 out of 650 packages were correctly classified by the classification software, resulting in a high accuracy rate of 99.08%. These results were obtained without any calibration on the system side, and several improvement and calibration processes were carried out to address the observed shortcomings. Primarily, it was considered that surface irregularities on certain parts of the packages placed on the conveyor prevented a flat surface from forming, leading to shadows or incomplete transmission of logo/color/text information to the camera, which in turn caused misclassifications. To prevent these issues, a positioning roller was integrated into the conveyor system to stabilize the packages as they pass through.

Additionally, improvements were made to stabilize ambient lighting, optimize the camera angle, and reconfigure image processing parameters (thresholds) according to the application environment. After these adjustments, the entire system was re-tested under active conditions. At this stage, speed/smoothness profiles (S-curve settings) of the robotic arm were reconfigured, and communication delays between the PLC and the robot were minimized.

To verify the operational structure of the system, a five-day production plan scenario was implemented. Each type of bag was processed at a rate of 4 bags per minute, aligned with a daily production rate of 1920 units. As shown in Table 16, three different bag types were sent to the system each day in sequence and were successfully classified and directed to their corresponding stacking areas. Some product groups were repeated on different days to observe system consistency. At the end of the five-day test, all 13 types of bags were successfully processed, classified, and stacked.

To evaluate the field performance of the developed system, a total of 9600 bag handling operations were monitored over a five-day production schedule. Each bag was classified by the image processing model, transmitted to the robot arm control unit via the PLC, and then directed by the robotic arm to its corresponding stacking point based on the identified class.

Following these comprehensive optimizations, the analysis showed that all bags were accurately detected, directed, and stacked without error. Based on this data, the system achieved a recorded overall routing accuracy of 100%.

### 4.3. Operational Performance Verification

During the five-day field test (9600 items), all classification and stacking events were automatically logged by the Siemens S7-1200 PLC and ABB controller. Each entry included class ID, timestamp, and completion signal from the robotic arm. No misclassification or misplacement events were recorded throughout the experiment, confirming 100% operational accuracy under the defined metric of “successful classification and stacking without interruption or rejection.” The logs also confirmed continuous synchronization between image inference, PLC signaling, and robotic motion, with no recorded communication or mechanical failures.

To protect against dust accumulation on the camera and optical components, the system was positioned inside a semi-enclosed metallic housing, which simultaneously reduces external reflections. As part of the preventive maintenance procedure, the lens surface is manually cleaned after every ≥120 h of operation. Throughout the 5-day continuous test (~80 h, ~9600 packages), no significant degradation was observed in image contrast or classification performance.

The conveyor line was subjected to 20–35 Hz mechanical oscillation with 0.8 mm amplitude; under these conditions, object detection remained stable, and no decrease in classification accuracy was detected.

In an industrial arrangement where the PLC and asynchronous motor VFD are located within the same control cabinet, the system was operated long-term. Under this challenging configuration, no misclassification, system freeze, or PLC–camera communication error occurred, and end-to-end communication latency remained below 3 ms. EMI impact is mitigated by the use of a metal camera enclosure, shielded Profinet cables, single-point grounding, and separation of power and signal lines.

This high success rate demonstrates that all system components including the classification model’s accuracy, the reliability of PLC data transmission, and the stable motion control of the robotic arm were optimized and operated in perfect synchronization. With this structure, the system meets the required levels of reliability and precision for industrial classification and automation applications.

## 5. Discussion

The YOLOv5-based real-time classification system developed in this study provides an effective and reliable solution for industrial package sorting. Throughout the experimental phase, several training configurations were evaluated to ensure the most practical and generalizable deployment. Specifically, both 80-20 and 70-15-15 stratified dataset splits were investigated, SGD and Adam optimizers were compared, and all YOLOv5 model scales (n, s, m, l, x) were tested under identical conditions.

Among these configurations, YOLOv5s trained with SGD at 640 × 640 resolution demonstrated the best balance of accuracy, stability, and real-time inference speed, making it the most appropriate model for industrial integration.

**Figure 16:**
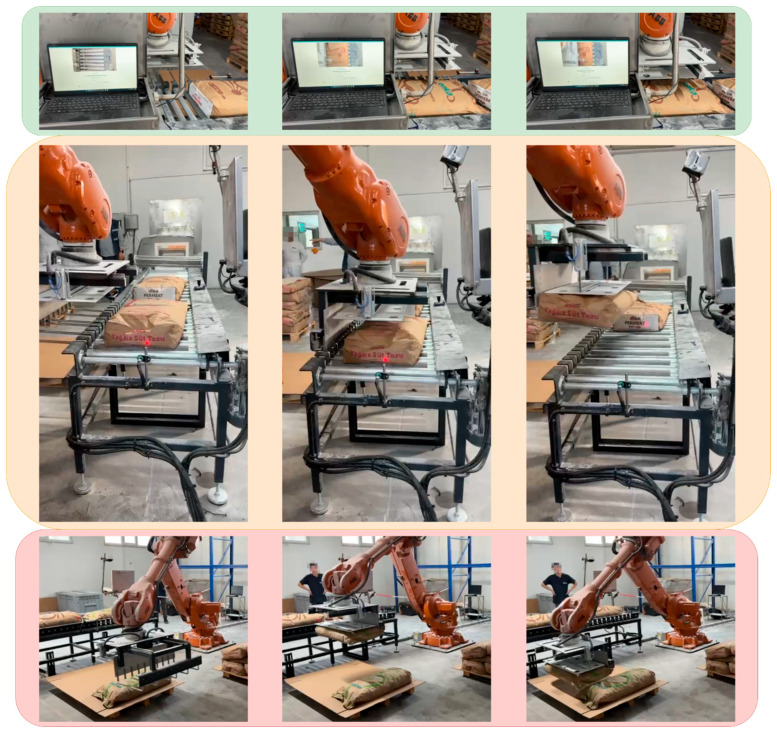
Images taken from the worksite where the system is actively operating.

This configuration achieved consistently high performance, with validation mAP@0.5 = 0.9849 and test mAP@0.5 = 0.9740, confirming reliable generalization to unseen samples. As observed in the class-wise results, nearly all classes maintained precision and recall above 97–99%, highlighting the system’s strong capability to distinguish visually similar packaging types in real-world conveyor environments. Images captured from the worksite where the system is actively operating are presented in Figure 16.

Although the initial training set consisted of 650 images, the risk of overfitting was mitigated through stochastic multi-transform augmentation, where images were exposed to different random augmentation combinations across epochs rather than a fixed replication of the dataset. The close alignment between validation and test results, as well as the model’s 100% operational success during the 9600-item industrial field run, further confirms that the performance reflects genuine applicability rather than memorization of the training data.

The complete operational cycle for a single package was also analyzed to quantify the real-time performance of the integrated perception-to-action pipeline. As triggered by the upstream proximity sensor (Sensor 1), image acquisition and preprocessing required approximately 0.25 s, followed by YOLOv5s GPU-based classification at 0.018 s per frame. The predicted class label was then transmitted to the robotic controller through an S7-1200 Profinet communication link in 0.20 s. The dominant portion of the cycle was the robotic pick–transfer–stack motion, requiring ~9.6 s using the S-curve trajectory plan. Overall, the fully synchronized framework achieved a cycle time of about 10–11 s per package, translating to an effective throughput of approximately six packages per minute, which satisfies real-time operational requirements for industrial conveyor-based stacking systems.

In addition to technical performance, the system was evaluated from an economic and scalability perspective. The total estimated investment, including hardware, software, and integration components such as the Siemens S7-1200 PLC, ABB IRB6640-130/3.2 robotic arm, Basler acA2500-14uc camera, and C125-0418-5M f/4 mm lens, is ≈55,000 USD. Compared to manual operation requiring two operators per shift, full automation is projected to reduce annual labor costs by approximately 70%. Assuming two shifts per day (16 h) for 300 working days per year, the estimated payback period is approximately 1.6 years. Furthermore, introducing new bag categories can be achieved efficiently via a structured workflow consisting of data collection → annotation → fine-tuning → validation → PLC parameter mapping → deployment → scheduled maintenance. Thus, the system is scalable and economically feasible for long-term industrial operation.

One of the system’s key advantages is its ability to make real-time decisions owing to its low-latency processing. With a total cycle time of approximately 10–11 s (including image processing, PLC communication, and robotic arm operation), the system meets the requirements of industrial standards and contributes to increased efficiency. Another major strength is its lightweight architecture, which enables operation on relatively low-spec hardware.

However, the system also has some limitations. For instance, certain classes such as Class8 exhibited slightly lower precision and mAP@0.5:0.95 values, suggesting that visual similarities or labeling inconsistencies may impact performance. It is important to note, though, that these minor metric drops did not negatively affect the system’s practical performance in real production scenarios. Observations in the field confirmed that Class8 items were still largely classified correctly, indicating that the system functions reliably in practice. This highlights that theoretical performance metrics do not always fully reflect real-world success, especially in cases where classes are visually similar. For such cases, a deeper analysis of the model’s decision making using attention map-based visualization techniques is planned to identify class-specific variations and improve performance through targeted data augmentation strategies. Additionally, future implementations may consider metric learning-based approaches to further enhance inter-class separability.

Beyond these points, while the system performs at a high level, it currently supports only the 13 predefined classes. Therefore, the introduction of new classes or variations would require retraining. However, this does not imply a lack of generalization ability. The architecture is flexible and open to new class integration. Owing to YOLOv5s’s compatibility with transfer learning, the model can be adapted to recognize new classes quickly by retraining only the final layers. The image processing software, labeling infrastructure, and augmentation methods allow new images to be processed efficiently, and, on the PLC side, stacking commands are defined parametrically by class ID making the system easily expandable without structural modification. In this regard, the system provides a flexible and sustainable foundation capable of adapting to future increases in product variety.

As alternative approaches, heavier models offering higher accuracy (e.g., YOLOv8-L or Faster R-CNN) could be considered, but these would increase processing time and compromise real-time performance. Similarly, models optimized for mobile platforms (e.g., YOLO-Nano) could enhance portability but may result in reduced classification accuracy. Therefore, it is essential to balance performance, speed, and cost based on the intended application of the system.

## 6. Conclusions and Future Work

Within the scope of this study, a real-time YOLOv5-based classification and robotic stacking system was developed for automated sorting of 13 different industrial bag types. Throughout the development process, multiple training configurations were explored, including 80-20 and 70-15-15 dataset splits, SGD vs. Adam optimizers, and different YOLOv5 model scales (n, s, m, l, x), to determine the most reliable and efficient deployment strategy. The selected configuration, YOLOv5s with SGD at 640 × 640 resolution, achieved strong overall performance, reaching mAP@0.5 = 0.9849 on the validation set and 0.9740 on the independent test set, confirming robust generalization to unseen samples.

These results translated effectively to industrial practice. During initial software-only validation in the facility, the system achieved 99.08% operational classification accuracy (644/650 items). After minor refinements, the fully integrated robotic stacking system was evaluated across 9600 continuously processed packages over five days, achieving 100% operational accuracy without misplacement or rejection. These outcomes demonstrate that the complete system has been effectively optimized and validated at both the perception and actuation levels.

Although the system employs standard modules such as YOLOv5s and the ABB IRB6640 robotic arm, its distinctiveness lies in the seamless synchronization and industrial communication design, enabling autonomous operation in a real production environment. This work bridges the gap between AI research and field-level Industry 4.0 deployment, transforming existing tools into an intelligent automation solution ready for practical use.

Although these accuracy values may appear high relative to typical object-detection benchmarks, they are attributable to the structured nature of the dataset and the stability of industrial imaging conditions. Each bag class exhibits distinctive visual identifiers (e.g., color, printing contrast, texture), and the imaging setup was optimized through controlled illumination, camera positioning, and mechanical alignment. Additionally, the employed augmentations enhanced the model’s tolerance to mild viewpoint and lighting variations. These factors collectively ensure reproducibility, practicality, and reliable performance in real-world applications.

The developed system also demonstrates operational flexibility, thanks to adjustable positioning elements, lighting alignment, and software configurability, allowing adaptation to different production lines with minimal modification. Future work will expand the number of supported packaging classes and integrate faster transfer learning to streamline adaptation to new product types. Moreover, deployment on edge AI platforms (e.g., Jetson Nano, Coral TPU) and development of a portable mobile interface are planned to further improve mobility and scalability.

## Figures and Tables

**Table 1:** RAPID motion commands and associated parameters.

Command	Target Point	Velocity	Zone
MoveJ	pStart	v150	z50
MoveL	pAboveBag	v120	z20
MoveL	pPick	v60	fine
MoveL	pLift	v120	z50
MoveJ	pStackApproach	v150	z100
MoveL	pStack	v60	fine

**Table 2:** Transformations and parameters used in the data augmentation process.

Transformation Type	Parameters
Rotation	Limit: ±30°, Probability: 0.7
Horizontal Flip	Probability: 0.5
Random Brightness & Contrast	Probability: 0.5 (default strength)
Gaussian Noise	Probability: 0.3
Motion Blur	Probability: 0.3 (default blur intensity)
Random Scale	Scale limit: ±10%, Probability: 0.4
Resize	640 × 640 (YOLO standard)

**Table 3:** Bag Class Descriptions.

Class	Dominant Color	Material Appearance	Distinctive Visual Features
1	Light brown	Kraft paper bag	Green circular label + dark purple bottom strip
2	Beige + brown	Kraft paper bag	Brown product image printed in center
3	Purple	Kraft paper bag	High-contrast white text + dark purple band
4	Dark blue	Kraft paper bag	Vertical orange stripe and logo
5	Red	Kraft paper bag	Bright red panel with white text
6	Light blue	Kraft paper bag	Light-blue panel + white contrast text
7	Beige	Kraft paper bag	Green curved marking (centered)
8	Grayish beige	Kraft paper bag	Low-contrast printed text—visually hardest class
9	Pink/red highlight	Kraft paper bag	Pink curved marking + bold contrast
10	Sky blue	Kraft paper bag	Blue vertical split background
11	Beige/green	Kraft paper bag	Light green vertical stripe
12	Beige/light green	Kraft paper bag	Green top band + circular badge
13	White	Kraft paper bag	Plain surface, minimal printing

**Table 4:** Technical training specifications and hyperparameters.

Features	Values
Input Image Size	640 × 640 Pixels
Number of Parameters	~7.5 million
Floating Point Operations (FLOPs)	~15.9 GFLOPs (for 640 × 640 input)
Number of Training Epochs	Maximum 100 (patience: 50)
Batch Size	16
Learning Strategy	Transfer Learning (yolov5s.pt)
Optimizers Evaluated	SGD, Adam
Selected Optimizer	SGD (momentum = 0.937, weight decay = 0.0005)
Learning Rate (LR)	(initial: 0.01–Final: 0.001)
Learning Rate Scheduler	Cosine Annealing (T_max_ = 100)
Loss Function	Complete IoU (CIoU)
Early Stopping	100 epochs completed

**Table 5:** Epoch-wise performance variations in YOLOv5s (80-20 split, SGD).

Epoch	Precision	Recall	mAP@0.5	mAP@0.5:0.95
0	0.21425	0.20462	0.09412	0.03514
1	0.37236	0.42400	0.41993	0.21016
2	0.44961	0.73465	0.67557	0.36371
3	0.52196	0.85713	0.77466	0.42942
4	0.86825	0.86116	0.90818	0.52911
5	0.90282	0.95206	0.96179	0.62819
10	0.93452	0.96589	0.98066	0.66708
20	0.95806	0.99077	0.98656	0.70831
30	0.97626	0.99385	0.99122	0.75893
40	0.98166	0.99692	0.99053	0.75957
50	0.97838	0.99692	0.99110	0.77378
60	0.98958	0.99692	0.99464	0.77984
70	0.98704	0.99538	0.99301	0.78100
80	0.99105	0.99385	0.99412	0.79006
90	0.99233	0.99344	0.99376	0.79281
98	0.99080	0.99164	0.99347	0.79937
99	0.98895	0.99231	0.99322	0.79909

**Table 6:** Epoch-wise performance variations in YOLOv5s (80-20 split, Adam).

Epoch	Precision	Recall	mAP@0.5	mAP@0.5:0.95
0	0.271775	0.238315	0.07476	0.024707
1	0.624055	0.259474	0.23524	0.10983
2	0.556345	0.49525	0.42945	0.22231
3	0.482345	0.66198	0.61341	0.28996
4	0.740165	0.64836	0.669245	0.328845
5	0.68497	0.792325	0.77988	0.41929
10	0.88332	0.885305	0.91303	0.536457
20	0.94293	0.933275	0.95518	0.59914
30	0.90747	0.949865	0.965805	0.634755
40	0.95315	0.964575	0.97431	0.654225
50	0.957285	0.976105	0.97797	0.68227
60	0.94656	0.985365	0.97665	0.702425
70	0.967745	0.98945	0.982325	0.711935
80	0.96869	0.987255	0.98243	0.72601
90	0.968275	0.987495	0.982735	0.738735
98	0.96961	0.982375	0.984845	0.745355
99	0.97021	0.98292	0.984285	0.74638

**Table 7:** Epoch-wise performance variations in YOLOv5s (80-20 split, AdaBoB).

Epoch	Precision	Recall	mAP@0.5	mAP@0.5:0.95
0	0.16611	0.67409	0.15924	0.092652
1	0.18291	0.75524	0.29436	0.1758
2	0.36011	0.69155	0.49438	0.29384
3	0.37584	0.84042	0.63869	0.3727
4	0.49849	0.81366	0.76296	0.50402
5	0.73193	0.82187	0.86156	0.56096
10	0.93334	0.96295	0.96587	0.61502
20	0.98582	0.98923	0.99248	0.73646
30	0.97629	0.99334	0.99154	0.73584
40	0.97992	0.99213	0.98912	0.74478
50	0.98236	0.9962	0.99145	0.76616
60	0.97918	0.99143	0.99058	0.75564
70	0.9794	0.99538	0.99008	0.75877
80	0.97805	0.99363	0.9909	0.77557
90	0.98357	0.99077	0.99139	0.77765
98	0.98082	0.99219	0.99093	0.78076
99	0.98092	0.99254	0.9892	0.7773

**Table 8:** Class-wise metrics of YOLOv5s (80-20 split, SGD).

Class	Precision	Recall	mAP@0.5	mAP@0.5:0.95
Class 1	0.995	1.0	0.995	0.845
Class 2	0.995	1.0	0.995	0.798
Class 3	0.994	1.0	0.995	0.777
Class 4	0.995	1.0	0.995	0.761
Class 5	0.995	1.0	0.995	0.826
Class 6	0.995	1.0	0.995	0.817
Class 7	0.993	1.0	0.995	0.839
Class 8	0.947	0.9	0.975	0.686
Class 9	0.994	1.0	0.995	0.875
Class 10	0.997	1.0	0.995	0.742
Class 11	0.993	1.0	0.995	0.867
Class 12	0.993	1.0	0.995	0.775
Class 13	0.994	1.0	0.995	0.794

**Table 9:** Test-set performance using different optimizers under the 80-20 split configuration.

Optimizer	Precision	Recall	mAP@0.5	mAP@0.5:0.95
SGD	0.989	0.992	0.993	0.799
AdaBoB	0.981	0.992	0.991	0.781
Adam	0.970	0.982	0.984	0.746

**Table 10:** Epoch-wise performance variations in YOLOv5s (70-15-15 split, SGD).

Epoch	Precision	Recall	mAP@0.5	mAP@0.5:0.95
0	0.21394	0.23	0.0741	0.02358
1	0.18436	0.34008	0.24133	0.094754
2	0.72926	0.47081	0.59937	0.28181
3	0.65485	0.78666	0.7583	0.33652
4	0.71832	0.76611	0.76391	0.35177
5	0.81456	0.82213	0.84706	0.4523
10	0.85587	0.917	0.91533	0.55325
20	0.94081	0.96019	0.97151	0.62854
30	0.95259	0.96904	0.9781	0.67992
40	0.95461	0.97996	0.98002	0.67719
50	0.96457	0.98385	0.98084	0.69765
60	0.97477	0.98653	0.98661	0.7169
70	0.97357	0.97324	0.98685	0.72758
80	0.97044	0.98678	0.9856	0.74352
90	0.97457	0.98242	0.98424	0.75033
98	0.97493	0.98073	0.98385	0.75429
99	0.9756	0.98026	0.9843	0.75325

**Table 11:** Epoch-wise performance variations in YOLOv5s (70-15-15 split, Adam).

Epoch	Precision	Recall	mAP@0.5	mAP@0.5:0.95
0	0.3293	0.26301	0.055399	0.013274
1	0.87575	0.078947	0.048474	0.0097008
2	0.66316	0.15385	0.17442	0.064752
3	0.45273	0.48283	0.45216	0.1685
4	0.61788	0.42663	0.43031	0.14858
5	0.47712	0.63259	0.59797	0.20439
10	0.83332	0.80472	0.8454	0.40231
20	0.9358	0.88778	0.9238	0.48997
30	0.83468	0.90388	0.94039	0.51058
40	0.93124	0.93623	0.95809	0.54888
50	0.94019	0.94729	0.96484	0.59076
60	0.90954	0.97381	0.95866	0.62501
70	0.94045	0.98382	0.97164	0.64287
80	0.93833	0.98126	0.97074	0.66196
90	0.94422	0.98235	0.97171	0.68466
98	0.95361	0.97351	0.97622	0.69134
99	0.95027	0.97349	0.97535	0.69367

**Table 12:** Epoch-wise performance variations in YOLOv5s (70-15-15 split, AdaBoB).

Epoch	Precision	Recall	mAP@0.5	mAP@0.5:0.95
0	0.31268	0.1753	0.08197	0.025788
1	0.18242	0.3125	0.20631	0.079182
2	0.31859	0.43709	0.39829	0.1663
3	0.30848	0.60439	0.39104	0.17696
4	0.53144	0.61319	0.58411	0.31369
5	0.51438	0.71762	0.70472	0.35644
10	0.93594	0.92897	0.95325	0.57553
20	0.93798	0.96233	0.9684	0.6148
30	0.95982	0.96549	0.97382	0.66713
40	0.95904	0.97885	0.97952	0.68051
50	0.95529	0.98585	0.97639	0.68676
60	0.95417	0.98821	0.98041	0.70103
70	0.97147	0.98403	0.98504	0.7104
80	0.95349	0.98091	0.97942	0.70734
90	0.95914	0.97178	0.9786	0.71599
98	0.96558	0.98573	0.98141	0.72828
99	0.96697	0.98434	0.97923	0.72139

**Table 13:** Class-wise metrics of YOLOv5s (70-15-15 split, SGD).

Class	Precision	Recall	mAP@0.5	mAP@0.5:0.95
Class 1	0.881	0.892	0.868	0.713
Class 2	0.991	1.0	0.995	0.831
Class 3	0.92	0.928	0.978	0.745
Class 4	0.997	1.0	0.995	0.701
Class 5	0.992	1.0	0.995	0.701
Class 6	0.99	1.0	0.995	0.683
Class 7	0.988	1.0	0.995	0.806
Class 8	0.8	0.838	0.882	0.644
Class 9	0.972	0.955	0.985	0.691
Class 10	0.997	1.0	0.995	0.637
Class 11	0.986	1.0	0.995	0.816
Class 12	0.986	1.0	0.995	0.834
Class 13	0.988	1.0	0.995	0.823

**Table 14:** Test-set performance using different optimizers under the 70-15-15 split configuration.

Optimizer	Precision	Recall	mAP@0.5	mAP@0.5:0.95
SGD	0.961	0.970	0.974	0.741
AdaBoB	0.960	0.976	0.972	0.714
Adam	0.950	0.969	0.965	0.679

**Table 15:** Real-time field test results of the software system.

Class ID	Number of Tested Packages	Correctly Classified Packages	Accuracy Rate (%)
1	50	50	100
2	50	50	100
3	50	49	98
4	50	50	100
5	50	50	100
6	50	50	100
7	50	50	100
8	50	47	94
9	50	49	98
10	50	49	98
11	50	50	100
12	50	50	100
13	50	50	100
Overall Accuracy Rate			99.08

**Table 16:** Real-time field test results of the fully integrated system.

Day	Class ID	Units per Product	Total Bags	Accuracy Rate (%)
1	1-2-3	640	1920	100
2	4-5-6	640	1920	100
3	7-8-9	640	1920	100
4	10-11-12	640	1920	100
5	13-1-2	640	1920	100
Overall Accuracy Rate				100

## Data Availability

The dataset used in this study is not publicly available due to industrial confidentiality constraints. However, it can be shared upon reasonable request from the authors for academic and non-commercial research purposes.

## Abbreviations

The following abbreviations are used in this manuscript:

YOLO	You Only Look Once
GAN	Generative Adversarial Network
AI	Artificial Intelligence
ANN	Artificial Neural Network
PLCs	Programmable Logic Controllers
CNN	Convolutional Neural Network
ROS	Robot Operating System
PANet	Path Aggregation Network
FLOPs	Floating Point Operations
TP	True Positive
FP	False Positive
FN	False Negative
TN	True Negative
IoU	Intersection over Union
mAP	Mean Average Precision
CIoU	Complete Intersection over Union
GUI	Graphical User Interface
